# Supplementary material for: A genomic appraisal of invasive Salmonella Typhimurium and associated antibiotic resistance in sub-Saharan Africa
Source: Nat Commun. 2023 Oct 23;14:6392. doi: 10.1038/s41467-023-41152-6 (PMC10593746; doi:10.1038/s41467-023-41152-6)
Supplement: Supplementary file 1 — Supplementary Information [file 41467_2023_41152_MOESM1_ESM.pdf]

A genomic appraisal of invasive *Salmonella* Typhimurium and associated antibiotic resistance in sub-Saharan Africa

Supplementary Figures

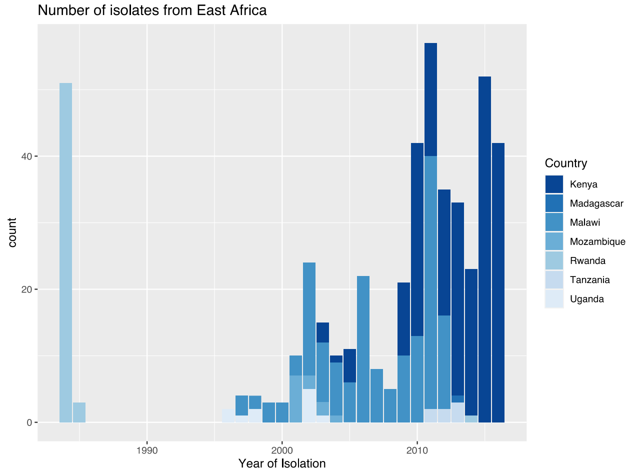

a

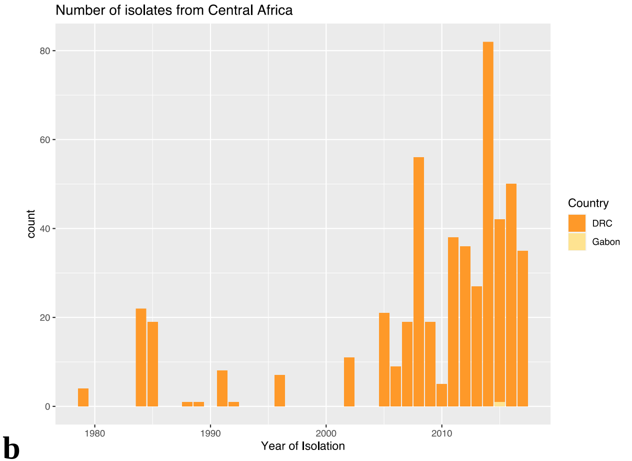

b

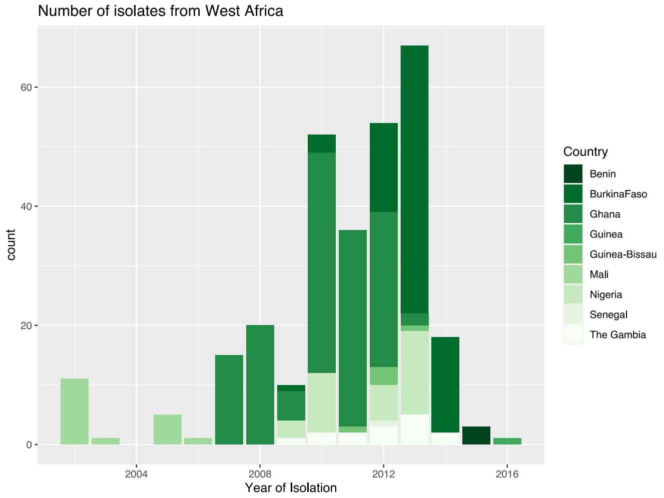

c

Supplementary Figure 1: *Salmonella* Typhimurium isolates included in this study per African region (a| East, b| Central and c| West) and year.

Isolates for which no year is known (NA, n = 15 African isolates) are not plotted.

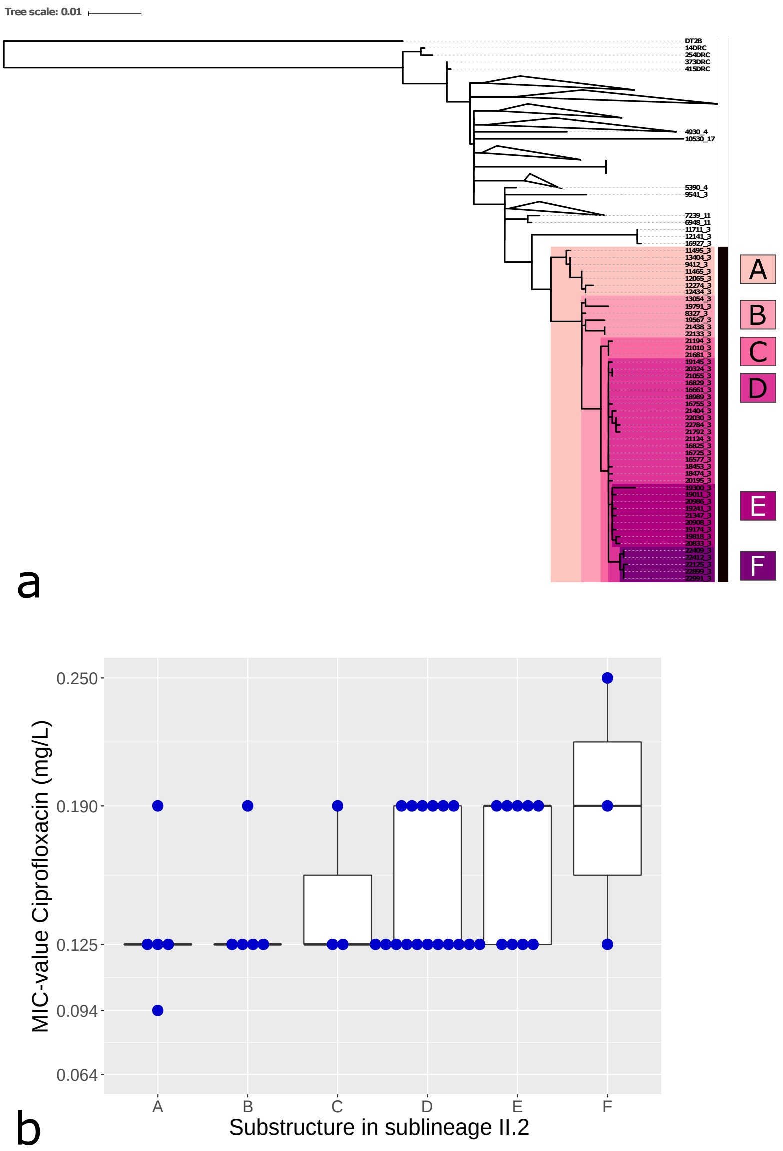

Supplementary Figure 2: Minimum inhibitory concentrations (MIC) for ciprofloxacin in nested substructures of invasive *Salmonella* Typhimurium clade ST313-L2 subclade 7 subclade showing quinolone resistance-determining region (QRDR) SNP underlying a GyrA S83Y substitution

a| Maximum likelihood phylogenetic tree of ST313-L2 *S.* Typhimurium isolates. Triangles indicate collapsed branches. The black strip indicates presence of the SNP coding for the GyrA S83Y substitution. Six substructures are annotated with different shades of pink. Mean MIC for substructure A = 0.132, B = 0.138, C = 0.147, D = 0.148, E = 0.161; F = 0.188 b| MIC ciprofloxacin values for n=42 independent isolates from ST313-L2 subclade 7, and grouped per substructure as coloured in pink in panel a. Boxplot centre lines represent median values, box limits present upper and lower quartiles; whiskers the 1.5 interquartile range and blue points the individual observations. The change of MIC over the nested substructures is found to be significant (p-value = 0.0235), and estimated to increase at 0.008695 mg/L of MIC per

nested substructure. This was tested using a two-sided generalized linear model (glm function) using R software, with a Gaussian distribution.

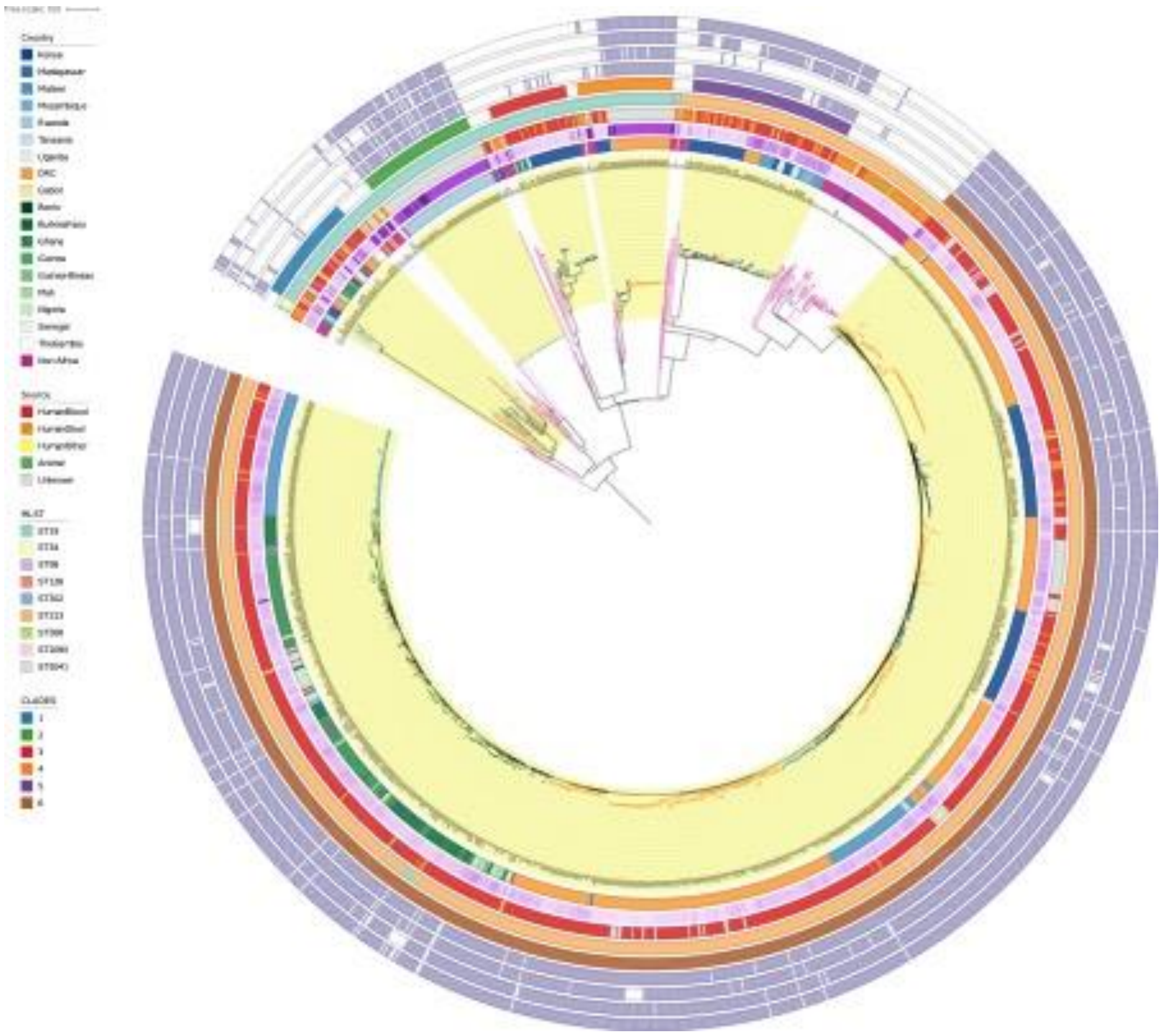

Supplementary Figure 3: The distribution of invasive *Salmonella* Typhimurium in Africa Maximum likelihood phylogenetic tree of the 1420 *S.* Typhimurium isolates sequences from this study (summarized in Supplementary Data 1). Sequencing reads were mapped to *S.* Typhimurium ST313 reference strain D23580. The tree is based on 71521 chromosomal SNPs. Branches are coloured by the country of isolation. Invasive *S.* Typhimurium clades as identified in this study are coloured in yellow (1=ST19-L1, 2=ST19-L2, 3=ST19-L3, 4=ST19-L4, 5=ST313-L1, 6=ST313-L2). Metadata is visualized on the concentric rings in compliance to the legend, from the inside to outside; 1. Country of origin, 2. Year of isolation as a gradient with darkest purple indicating the oldest isolates, 3. Source of isolation, 4. MLST, 5. Clade, 6-9. Presence of multidrug resistance markers (*MDR; blaTEM, cat, dfrA*). Branch lengths represent the number of SNPs as indicated in the scale bar.

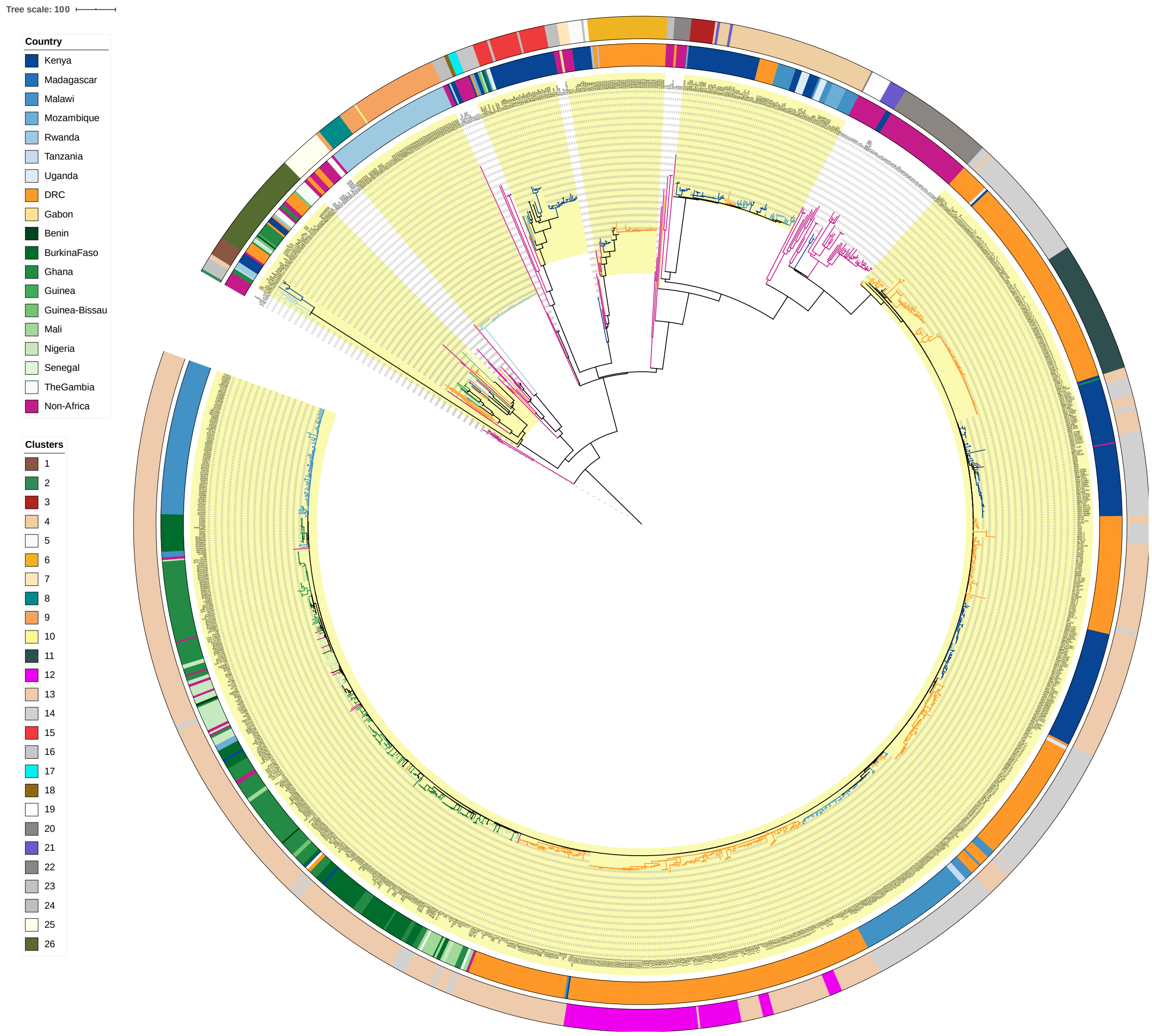

Supplementary Figure 4: Classification of *Salmonella* Typhimurium in clades using HierBaps clusters.

Maximum likelihood phylogenetic tree of the 1420 *S.* Typhimurium isolates sequences from this study. Metadata is visualized on the concentric rings in compliance to the legend, from the inside to outside; 1. Country of origin, 2. The second level of HierBaps clusters resulting from analysis of the full dataset (n =1420). These clusters were used to define the invasive *S.* Typhimurium clusters and are plotted on the outer circle. Six clades were identified, based on respectively Hierbaps cluster 1, 26 (ST19-L1, 115 SNPs, n = 56), 8, 9, 10 (ST19-L2, 214 SNPs, n = 63), 15, 16 (ST19-L3, 67 SNPs, n = 42), 5, 6 , 7 (ST19-L4, 101 SNPs, n = 51), 3, 4 (ST313-L1, 192 SNPs, n = 87), 11, 12, 13, 14 (ST313-L2, 72 SNPs, n = 1005). The inner circle and branches are coloured by the country of origin. Branch lengths represent the number of SNPs as indicated in the scale bar.

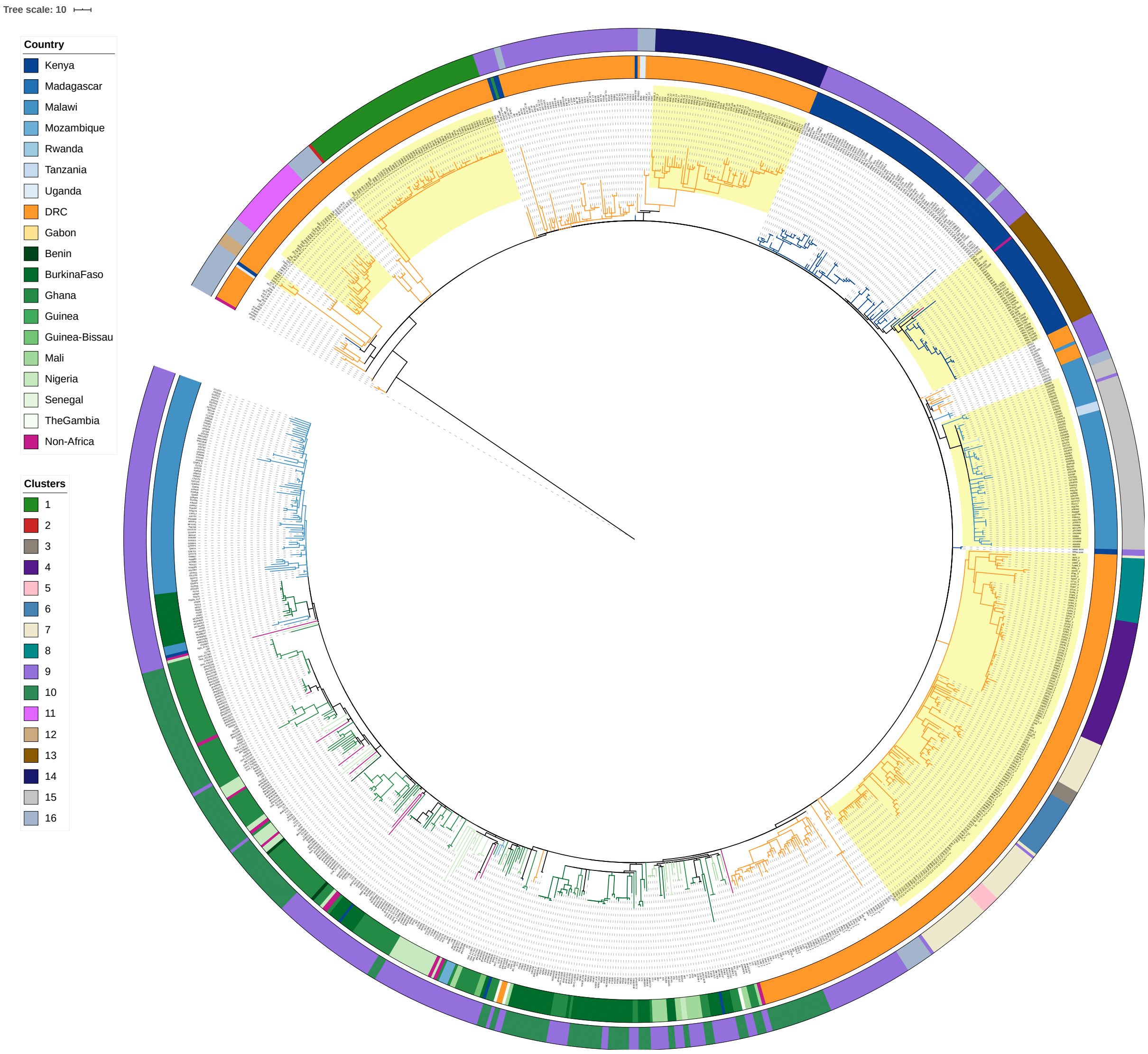

Supplementary Figure 5: Classification of *Salmonella* Typhimurium ST313-L2 in subclades using HierBaps clusters.

Maximum likelihood phylogenetic tree of ST313-L2 isolates. The second level of HierBaps clusters resulting from the analysis of the ST313-L2 isolates (n = 1006) were used to define the ST313-L2 subclusters and are plotted on the outer circle. Seven subclades were identified, based on respectively Hierbaps cluster 12 (subclade 1, 50 SNPs, n = 4), 11 (subclade 2, 15 SNPs, n = 24), 1, 2 (subclade 3, 14 SNPs, n = 61), 15 (subclade 4, 10 SNPs, n = 57), 13 (subclade 5, 5 SNPs, n = 39), 15 (subclade 6, 4 SNPs, n = 63), 4, 5, 6, 7, 8 (subclade 7, 8 SNPs, n = 150). The inner circle and branches are coloured by the country of origin. Branch lengths represent the number of SNPs as indicated in the scale bar.

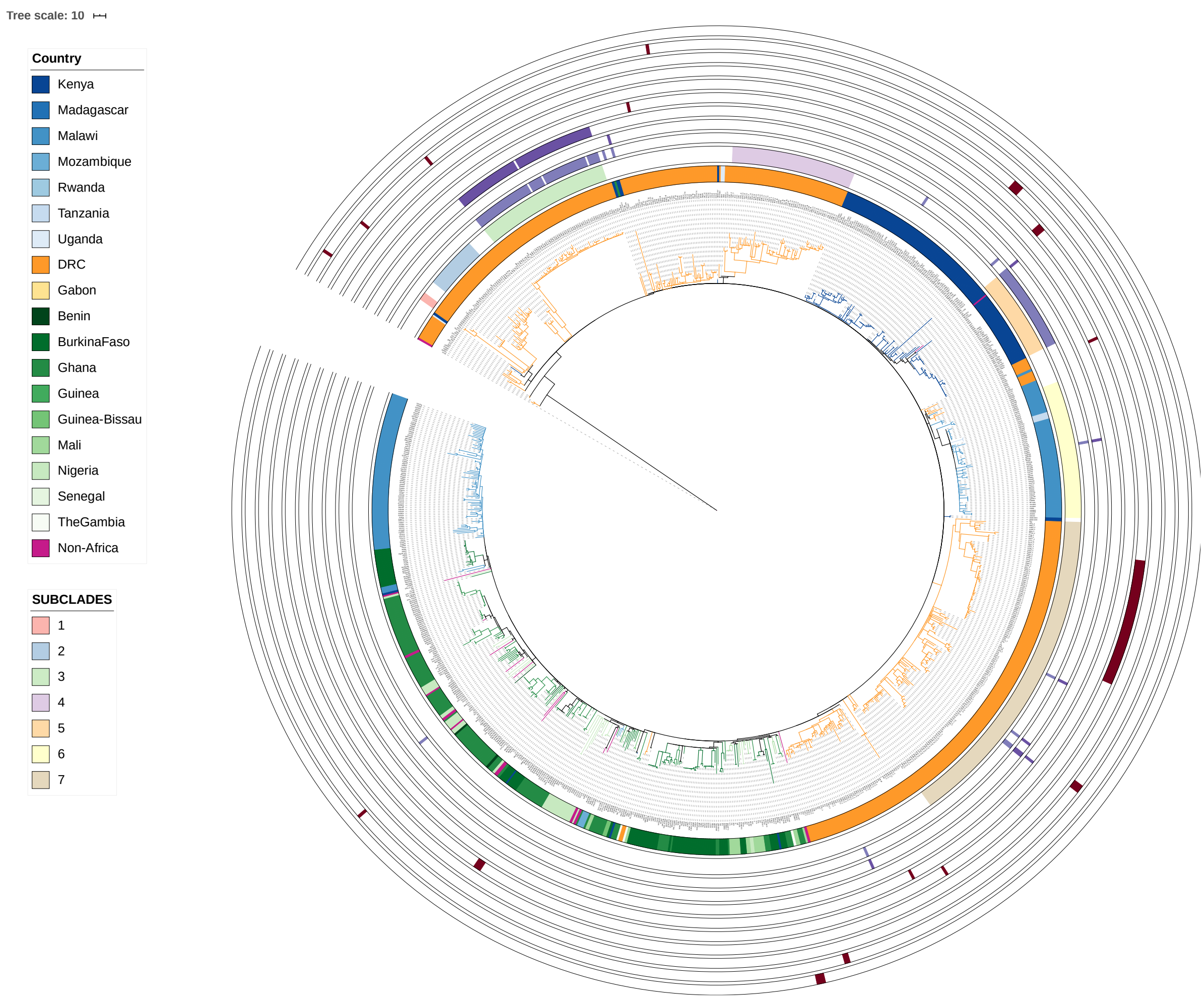

Supplementary Figure 6: Genetic markers for extended spectrum beta lactamase (ESBL) activity, fluoroquinolone resistance, azithromycin resistance and quinolone resistance determining regions (QRDR) SNPs in *S.* Typhimurium ST313-L2.
Maximum likelihood phylogenetic tree of ST313-L2 isolates. The inner two circles indicate the country of isolation and the iTYM6 subclade. The sequential rings indicate presence of (from the inside to the outside): ESBL, fluoroquinolone resistance marker (*qnr*), azithromycin resistance marker (*mphA*), GyrA S83F, GyrA S83Y, GyrA D87G, GyrA D87N, GyrA D87Y, GyrB E466D. Branch lengths represent the number of SNPs as indicated in the scale bar.

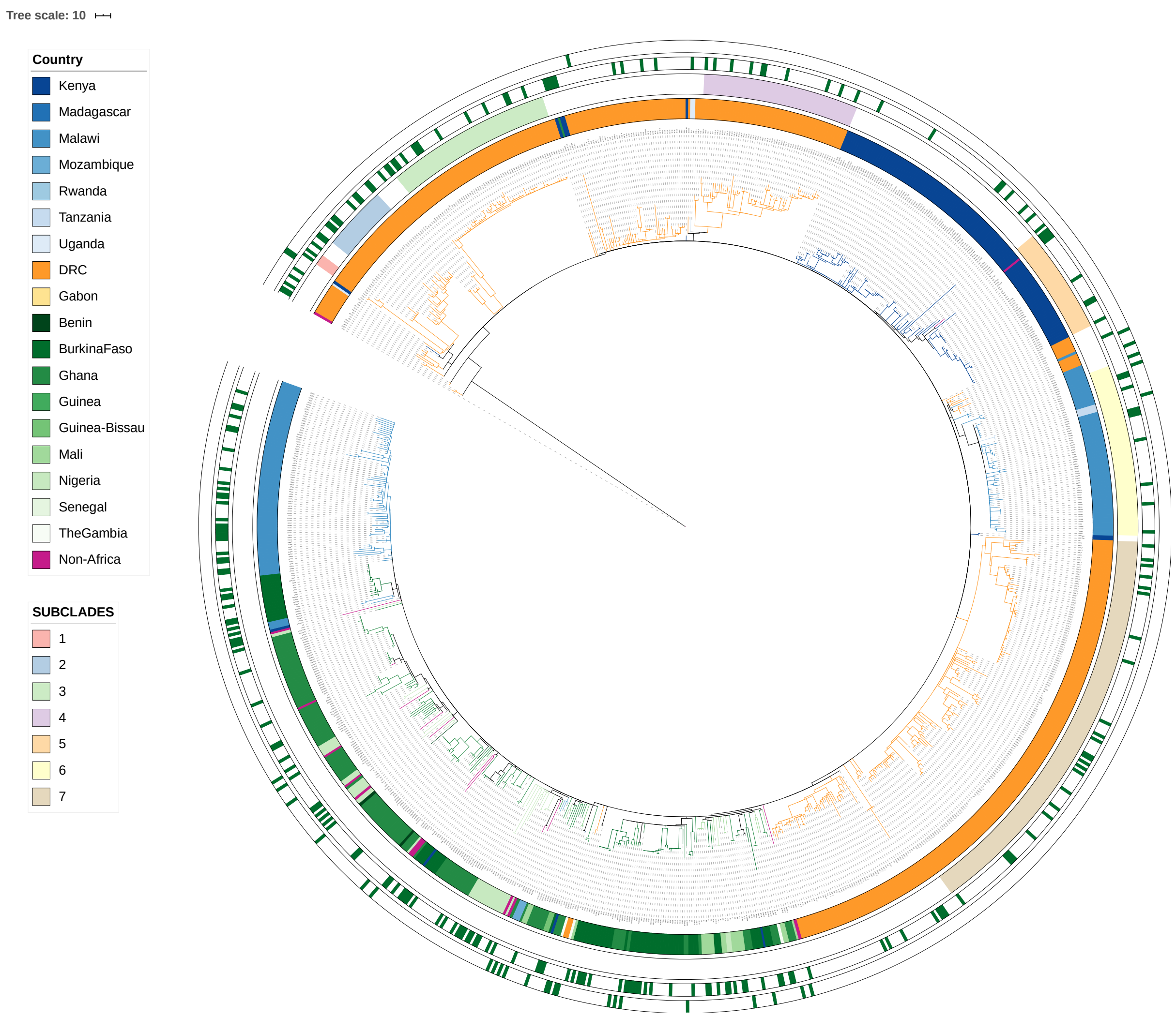

Supplementary Figure 7: Selection of isolates used for the phylogeographical analysis Maximum likelihood phylogenetic tree of ST313-L2 isolates. The inner two circles indicate the country of isolation and the ST313-L2 subclade. The outer two rings indicate the isolates selected for the spatiogeographical analysis. The third circle is the selection made by CD-hit (n=220) and the outer circle is a selection including all additional geographical events (n = 31). In addition, strain *S.* Typhimurium D23580 was added to the set of isolates.

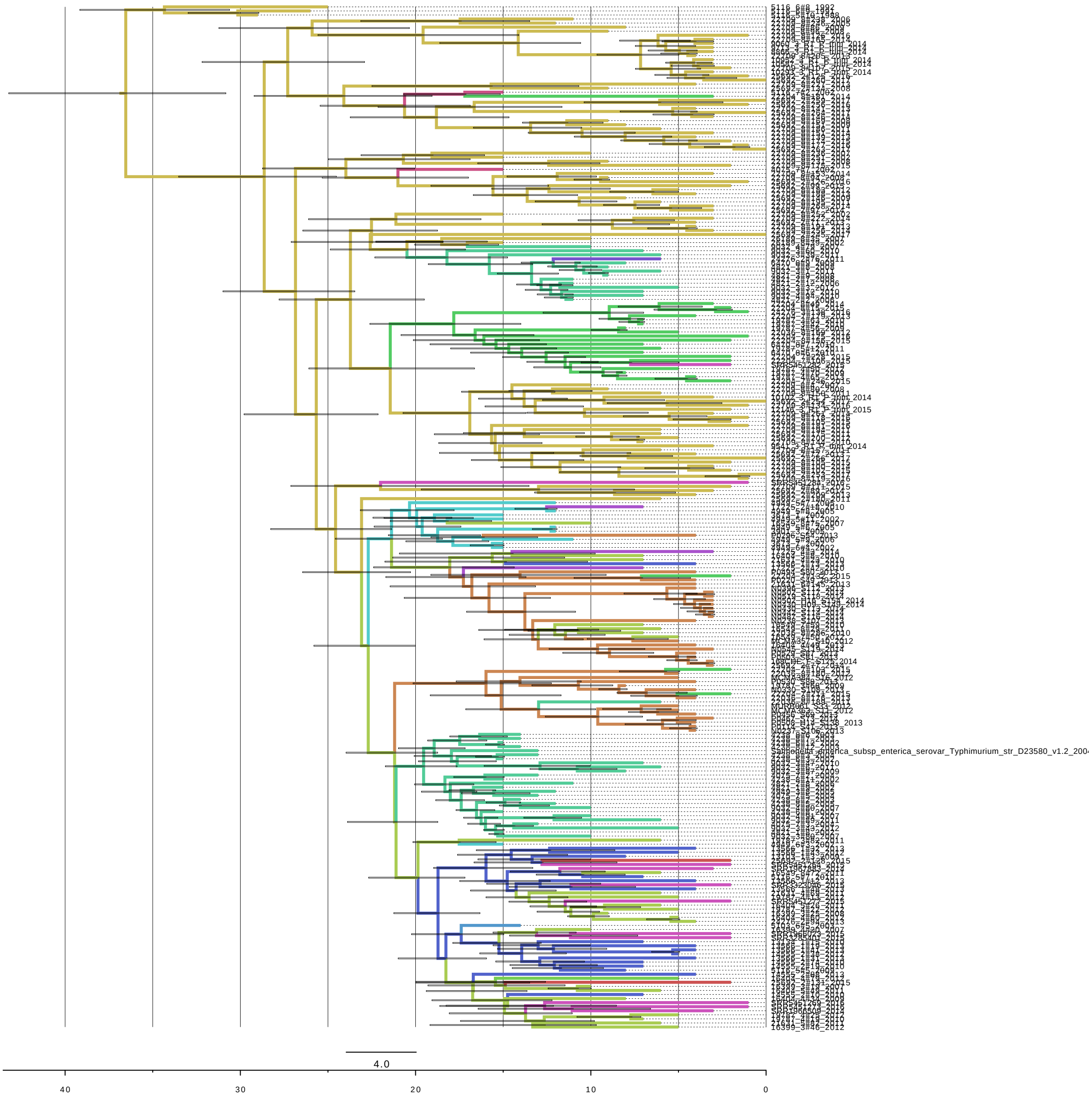

Supplementary Figure 8: Time-tree from BEAST showing the phylogeographical reconstruction of ST313-L2

Raw tree underlying figure 3A including the isolate labels and the 95% HPD intervals.

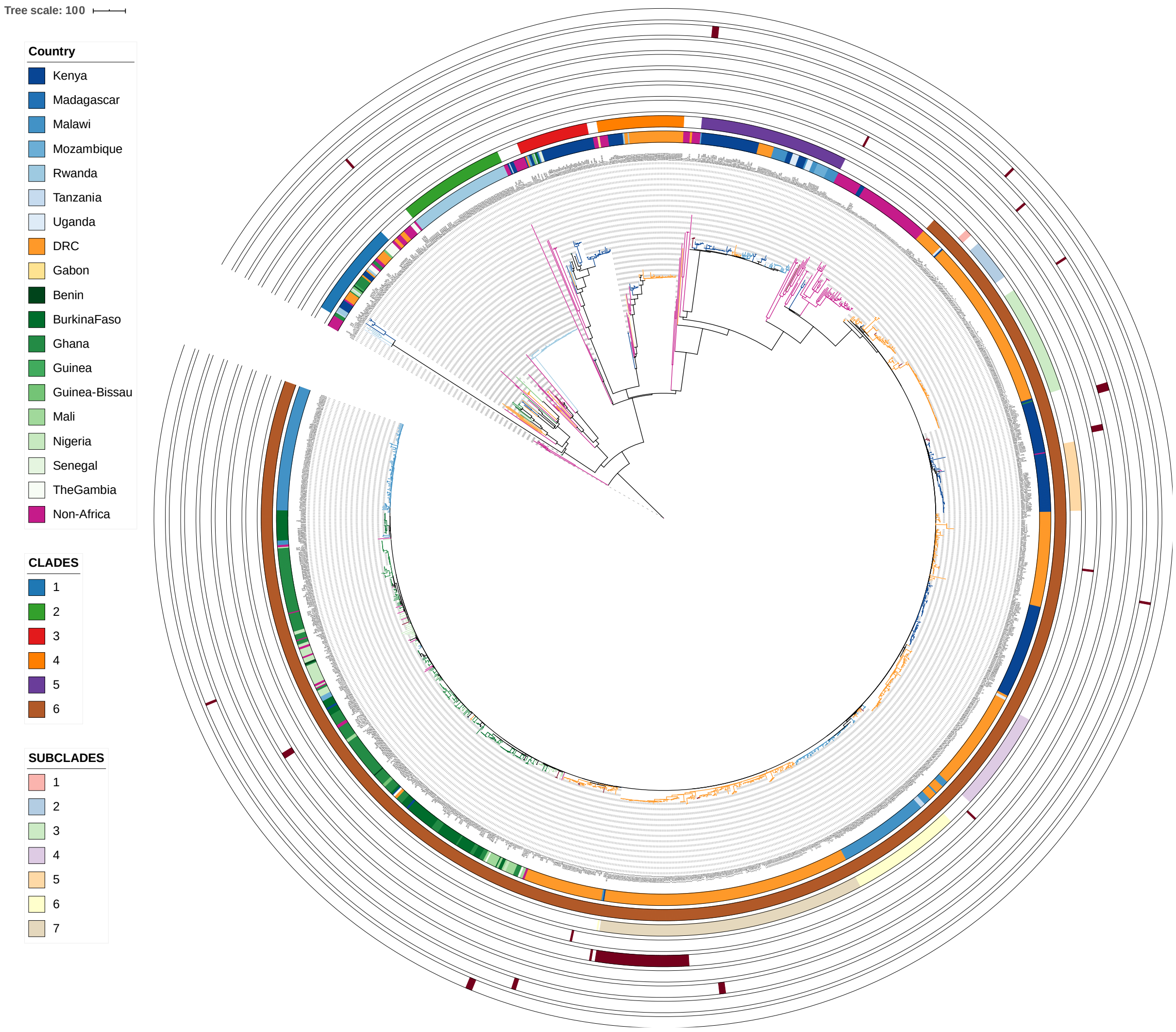

Supplementary Figure 9: Quinolone resistance-determining regions in *Salmonella* Typhimurium

Maximum likelihood phylogenetic tree of the 1420 *S.* Typhimurium isolates sequences from this study. The inner three circles indicate the country of isolation, the invasive *S.* Typhimurium clade and the iTYM6 subclade. The sequential rings indicate presence of subsitutions (from the inside to the outside): GyrA S83F, GyrA S83Y, GyrA D87G, GyrA D87N, GyrA D87Y, GyrB E466D

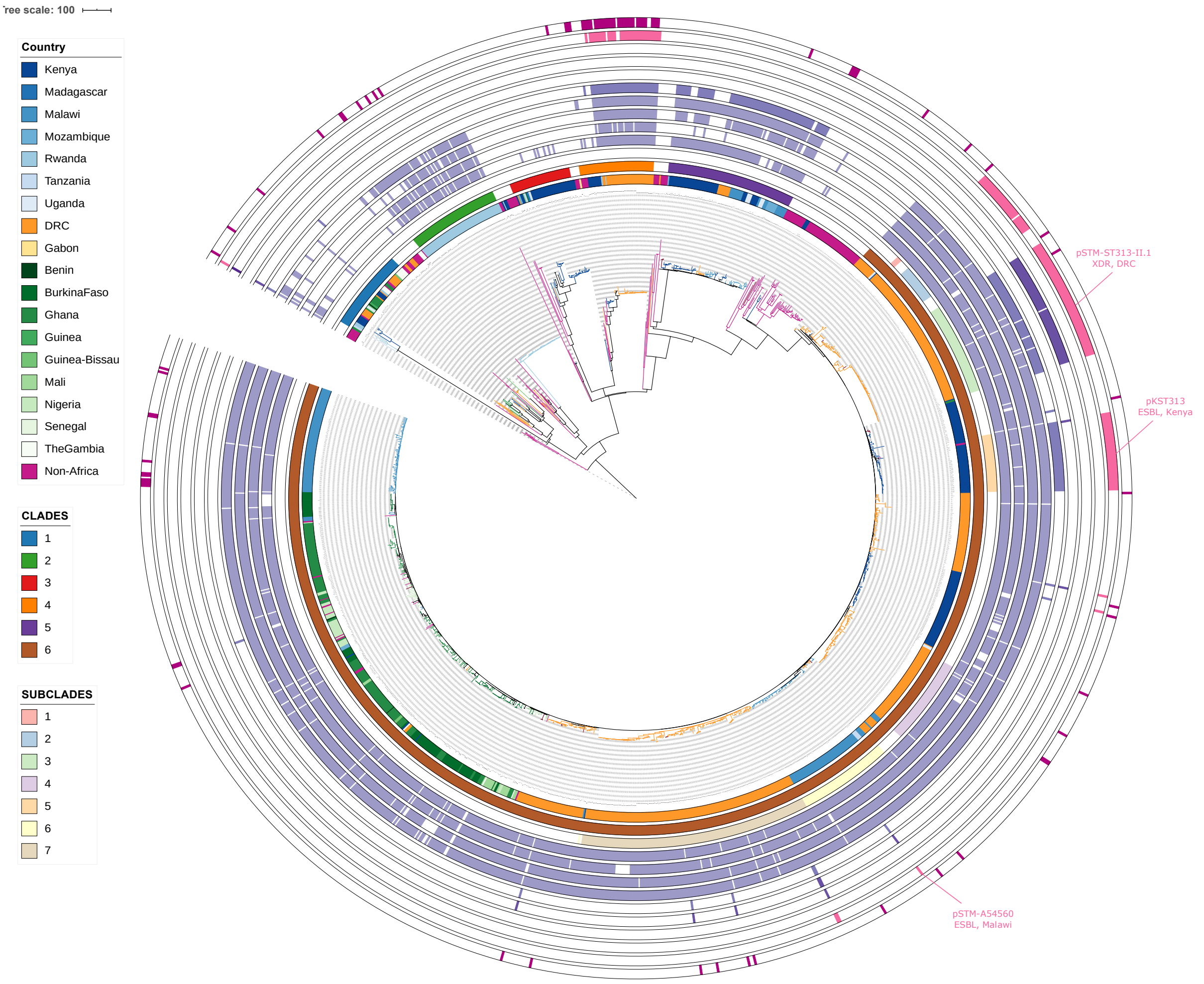

Supplemental Figure 10: IncI1 and IncHI2 plasmids driving XDR and PDR

Maximum likelihood phylogenetic tree of the 1420 *S.* Typhimurium isolates sequences from this study. Metadata is visualized on the concentric rings, from the inside to outside; 1. Country of origin, 2. Invasive *S.* Typhimurium clades, 3. ST313-L2 subclades, 4-7. Presence of multidrug resistance markers (MDR; *blaTEM*, *cat*, *dfrA*, *sul*), 8-10. Presence of extensively and pan drug resistance markers (XDR and PDR; *ESBL*, *qnr*, *mphA*), 11. Presence of colistin resistance (*mcr*), 12-13. Presence of IncHI2 and IncHI1 plasmid replicons. Branch lengths represent the number of SNPs as indicated in the scale bar. The XDR plasmid pSTM-ST313-II.1 (DRC, Van Puyvelde et al, 2019) and ESBL plasmids pKST313 (Kenya, Kariuki et al, 2015) and pSTM-A54560 (Malawi, Feasey et al, 2014) have been annotated on the tree figure.

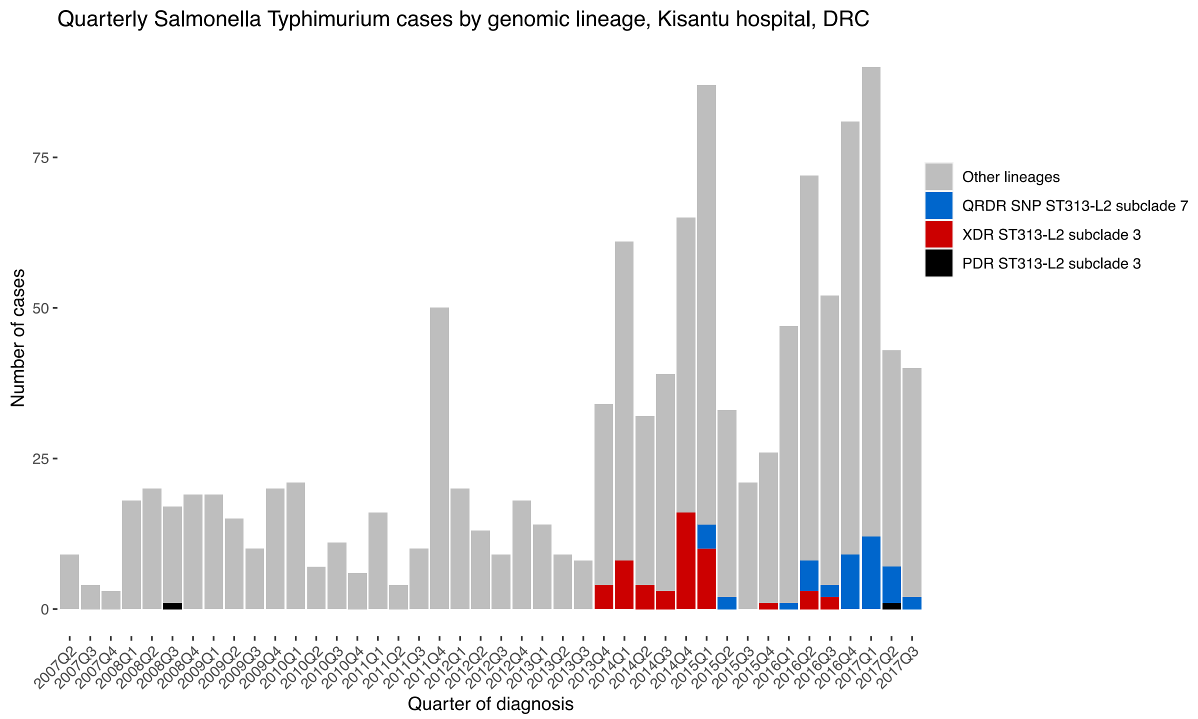

Supplementary Figure 11: Temporal evolution of *Salmonella* Typhimurium outbreaks in Kisantu, DRC, 2007-2017

Number of invasive *S.* Typhimurium cases per quarter. Isolates belonging to extensively drug resistance (XDR) ST313-L2 subclade 3 clade and the ST313-L2 subclade 7 clade containing quinolone resistance-determining region (QRDR) SNPs are coloured. Two isolates of the ST313-L2 subclade 3 clade presented pan drug resistance (PDR) and are coloured differentially.

Supplementary Tables

Supplementary Table 1: Genes presenting non-synonymous mutations in at least two invasive *S.* Typhimurium clades

Single nucleotide polymorphisms between isolates in each invasive *S.* Typhimurium clade and context isolates were extracted and annotated as their effect in the *S.* Typhimurium SL1344 genome sequence. Genes with non-synonymous mutations were identified and genes found in at least two different clades are listed.

Clade	Higher level function	Gene name	Function
ST19-L2, ST19-L3, ST313-L1	metabolism	*malZ*	Maltodextrin glucosidase. In *E. coli*, loss of *malZ* results in degradation of maltodextrins to maltotriose [1, 2]. It has not been recognized previously as a pseudogene.
ST19-L3, ST313-L1, ST313-L2	host interactions/virulence	*ssrA*	Two-component sensor kinase encoded on SPI-2, involved in regulation of pathogenesis through sensing of Ca^{2+} and acidic environments [3, 4].
ST19-L4, ST313-L1, ST313-L2	membrane/surface-associated	*stfC*	Outer membrane usher protein (hypothetical fimbrial outer membrane usher) implicated in persistence of *Salmonella* on lettuce. Observed as a pseudogene in endosymbiont *Sodalis glossinidius* [5, 6].
ST19-L1, ST19-L2	other	*ygcH*	Conserved hypothetical protein [7].
	cell division	*mukB*	Subunit of the condensing complex in *Salmonella* and *E. coli* that is involved in compacting chromosomal DNA. No prior evidence of pseudogenization [8].
ST19-L1, ST19-L3	redox-associated	*ydiJ*	D-2-hydroxyglutarate dehydrogenase in *Enterobacteriaceae* [9].
ST19-L1, ST19-L4	phage	SL1344_2600	Predicted bacteriophage integrase [10]
ST19-L2, ST19-L3	membrane/surface-associated	*dacD*	Penicillin-binding protein involved in removal of D-alanyl restudies from cell wall building blocks. Loss of dacD in *Salmonella enterica* results in greater susceptibility to cefotaxime and altered biofilm growth [11]
	membrane/surface-associated	*fimA*	Type-1 fimbrial protein, major subunit, involved in cellular adhesion. Loss of *fimA* in *S.* Typhimurium results in inability to assemble fimbriae [12, 13]. Pseudogene previously observed in *Bordetella pertussis* [14]
	other	*ydiD*	Cyclohexanecarboxylate-CoA ligase involved in intestinal butyrate metabolism. Pseudogenization observed in *S.* Typhi, and *ydiD* loss in *S.* Typhimurium is implicated in lower epithelial invasion [15, 16].
	redox-associated	*ccmH2*	Cytochrome C-type biogenesis protein H2[10]
	transport	SL1344_0377	MFS transporter; hypothetical efflux pump[10]

ST19-L2, ST19-L4	motility	*fliF*	Transmembrane flagellar basal-body M-ring protein [17]. Partial deletion of *fliF* is known to affect flagellar rotation directionality.
ST19-L2, ST313-L1	redox-associated	*Bcp*	bacterioferritin comigratory protein, implicated in hydrogen peroxidate scavenging [17, 18, 19]
ST19-L2, ST313-L2	other	SL1344_1551	Hypothetical exported protein [10]
	metabolism	*pdxK*	Pyridoxine kinase involved in vitamin B6 metabolism [20]
ST19-L3, ST19-L4	transcription	*sbcC*	Exonuclease for homologous recombination. Mutation of *sbcC* (or *sbcB*) activates the RecF recombination pathway for DNA repair [21, 22].
	metabolism	*eutA*	Reactivase for ethanolamine utilization; loss of *eutA* limits ethanolamine metabolism to when adenosyl-B12 is available [23, 24]
ST19-L3, ST313-L1	nucleoid-associated	SL1344_2552	(Proposed) Glucans biosynthesis protein D involved in structure of periplasmic glucans
	host interactions/virulence	*shdA*	Fibronectin-binding outer membrane protein. Previously found to be pseudogenenized in *S.* Typhi, *S.* Paratyphi, and other host-adapted serovars [25].
	nucleoid-associated	*hrpA*	ATP-dependent RNA helicase [10]
	other	SL1344_1983	hypothetical exported protein [10]
	redox-associated	SL1344_1475	Hypothetical alkyl hydroperoxide reductase subunit F [10]
ST19-L3, ST313-L2	other	*cutC*	Recognized as choline TMA-lyase in gut commensal bacteria involved in anaerobic choline utilization, though specific function in *Salmonella* has not been identified [26]
	host interactions/virulence	*steC*	Type III secretion system effector protein found to be a pseudogene in passerine-adapted *S.* Typhimurium, associated with lower invasion but higher virulence in hosts [27, 28].
	phage	SL1344_2582	Predicted DUF1367 family bacteriophage protein [10]

References

1. Schneider E, Freundlieb S, Tapio S, Boos W. Molecular characterization of the MalT-dependent periplasmic alpha-amylase of Escherichia coli encoded by malS. *J Biol Chem* **267**, 5148-5154 (1992).

2. Dippel R, Boos W. The maltodextrin system of Escherichia coli: metabolism and transport. *J Bacteriol* **187**, 8322-8331 (2005).

3. Julio SM, Heithoff DM, Mahan MJ. ssrA (tmRNA) plays a role in Salmonella enterica serovar Typhimurium pathogenesis. *J Bacteriol* **182**, 1558-1563 (2000).

4. Garmendia J, Beuzon CR, Ruiz-Albert J, Holden DW. The roles of SsrA-SsrB and OmpR-EnvZ in the regulation of genes encoding the Salmonella typhimurium SPI-2 type III secretion system. *Microbiology (Reading)* **149**, 2385-2396 (2003).

5. Goodhead I, *et al.* Large-scale and significant expression from pseudogenes in Sodalis glossinidius - a facultative bacterial endosymbiont. *Microb Genom* **6**, (2020).

6. Kroupitski Y, *et al.* Identification of Salmonella enterica genes with a role in persistence on lettuce leaves during cold storage by recombinase-based in vivo expression technology. *Phytopathology* **103**, 362-372 (2013).

7. Ebihara A, Yao M, Masui R, Tanaka I, Yokoyama S, Kuramitsu S. Crystal structure of hypothetical protein TTHB192 from Thermus thermophilus HB8 reveals a new protein family with an RNA recognition motif-like domain. *Protein Sci* **15**, 1494-1499 (2006).

8. Rovinskiy NS, Agbleke AA, Chesnokova ON, Higgins NP. Supercoil Levels in E. coli and Salmonella Chromosomes Are Regulated by the C-Terminal 35(-)38 Amino Acids of GyrA. *Microorganisms* **7**, (2019).

9. Samsonov VV, Kuznetsova AA, Rostova JG, Samsonova SA, Ziyatdinov MK, Kiriukhin MY. Revealing a New Family of D-2-Hydroxyglutarate Dehydrogenases in Escherichia coli and Pantoea ananatis Encoded by ydiJ. *Microorganisms* **10**, (2022).

10. Kroger C, *et al.* The transcriptional landscape and small RNAs of Salmonella enterica serovar Typhimurium. *Proc Natl Acad Sci U S A* **109**, E1277-1286 (2012).

11. Brambilla L, Moran-Barrio J, Viale AM. Low-molecular-mass penicillin binding protein 6b (DacD) is required for efficient GOB-18 metallo-beta-lactamase biogenesis in Salmonella enterica and Escherichia coli. *Antimicrob Agents Chemother* **58**, 205-211 (2014).

12. Duncan MJ, Mann EL, Cohen MS, Ofek I, Sharon N, Abraham SN. The distinct binding specificities exhibited by enterobacterial type 1 fimbriae are determined by their fimbrial shafts. *J Biol Chem* **280**, 37707-37716 (2005).

13. Zeiner SA, Dwyer BE, Clegg S. FimA, FimF, and FimH are necessary for assembly of type 1 fimbriae on Salmonella enterica serovar Typhimurium. *Infect Immun* **80**, 3289-3296 (2012).

14. Willems RJ, van der Heide HG, Mooi FR. Characterization of a Bordetella pertussis fimbrial gene cluster which is located directly downstream of the filamentous haemagglutinin gene. *Mol Microbiol* **6**, 2661-2671 (1992).

15. Bronner DN, *et al.* Genetic Ablation of Butyrate Utilization Attenuates Gastrointestinal Salmonella Disease. *Cell Host Microbe* **23**, 266-273 e264 (2018).

16. Campbell JW, Morgan-Kiss RM, Cronan JE, Jr. A new Escherichia coli metabolic competency: growth on fatty acids by a novel anaerobic beta-oxidation pathway. *Mol Microbiol* **47**, 793-805 (2003).

17. Sakai T, *et al.* Novel Insights into Conformational Rearrangements of the Bacterial Flagellar Switch Complex. *mBio* **10**, (2019).

18. Mishra S, Imlay J. Why do bacteria use so many enzymes to scavenge hydrogen peroxide? *Arch Biochem Biophys* **525**, 145-160 (2012).

19. Reeves SA, Parsonage D, Nelson KJ, Poole LB. Kinetic and thermodynamic features reveal that Escherichia coli BCP is an unusually versatile peroxiredoxin. *Biochemistry* **50**, 8970-8981 (2011).

20. Yang Y, Zhao G, Winkler ME. Identification of the pdxK gene that encodes pyridoxine (vitamin B6) kinase in Escherichia coli K-12. *FEMS Microbiol Lett* **141**, 89-95 (1996).

21. Bidnenko V, Seigneur M, Penel-Colin M, Bouton MF, Dusko Ehrlich S, Michel B. sbcB sbcC null mutations allow RecF-mediated repair of arrested replication forks in rep recBC mutants. *Mol Microbiol* **33**, 846-857 (1999).

22. Galitski T, Roth JR. Pathways for homologous recombination between chromosomal direct repeats in Salmonella typhimurium. *Genetics* **146**, 751-767 (1997).

23. Roof DM, Roth JR. Functions required for vitamin B12-dependent ethanolamine utilization in Salmonella typhimurium. *J Bacteriol* **171**, 3316-3323 (1989).

24. Costa FG, Escalante-Semerena JC. Localization and interaction studies of the Salmonella enterica ethanolamine ammonia-lyase (EutBC), its reactivase (EutA), and the EutT corrinoid adenosyltransferase. *Mol Microbiol* **118**, 191-207 (2022).

25. Betancor L, *et al.* Genomic Comparison of the Closely Related Salmonella enterica Serovars Enteritidis and Dublin. *Open Microbiol J* **6**, 5-13 (2012).

26. Craciun S, Marks JA, Balskus EP. Characterization of choline trimethylamine-lyase expands the chemistry of glycyl radical enzymes. *ACS Chem Biol* **9**, 1408-1413 (2014).

27. Fu Y, Smith JC, Shariat NW, M'Ikanatha N M, Dudley EG. Evidence for common ancestry and microevolution of passerine-adapted Salmonella enterica serovar Typhimurium in the UK and USA. *Microb Genom* **8**, (2022).

28. Cohen E, *et al.* Pathoadaptation of the passerine-associated Salmonella enterica serovar Typhimurium lineage to the avian host. *PLoS Pathog* **17**, e1009451 (2021).
