# Supplementary material for: A genomic appraisal of invasive Salmonella Typhimurium and associated antibiotic resistance in sub-Saharan Africa
Source: Nat Commun. 2023 Oct 23;14:6392. doi: 10.1038/s41467-023-41152-6 (PMC10593746; doi:10.1038/s41467-023-41152-6)
Supplement: Supplementary file 3 — Description of Additional Supplementary Files [file 41467_2023_41152_MOESM3_ESM.pdf]

Description of Additional Supplementary Files

Supplementary Data 1: Overview of 1420 *S.* Typhimurium isolates used in this study. The respective metadata, accession IDs and clade assignments are given per isolate.

Supplementary Data 2: Quality statistics of assemblies per isolate.

Supplementary Data 3: List of 7 isolates subjected to Nanopore sequencing.
The respective metadata and accession IDs are given per isolate. Per isolate contigs are listed with their nucleotide length. Chromosomal contigs are coloured in blue, whereas plasmid contigs are coloured in orange. For plasmid contigs, the plasmid type is included.

Supplementary Data 4: List of 60 isolates from this study used in the *S.* Typhimurium context analysis, comprising ten isolates per invasive *S*. Typhimurium clade.

Supplementary Data 5: List of 131 additional *S.* Typhimurium isolates used for the context analysis. The accession ID per isolate is given.
