# Supplementary material for: A genomic appraisal of invasive Salmonella Typhimurium and associated antibiotic resistance in sub-Saharan Africa
Source: Nat Commun. 2023 Oct 23;14:6392. doi: 10.1038/s41467-023-41152-6 (PMC10593746; doi:10.1038/s41467-023-41152-6)
Supplement: Supplementary file 9 — Reporting Summary [file 41467_2023_41152_MOESM9_ESM.pdf]

nature portfolio

Corresponding author(s): Sandra Van Puyvelde

Last updated by author(s): Jun 29, 2023

Reporting Summary

Nature Portfolio wishes to improve the reproducibility of the work that we publish. This form provides structure for consistency and transparency in reporting. For further information on Nature Portfolio policies, see our Editorial Policies and the Editorial Policy Checklist.

Statistics

For all statistical analyses, confirm that the following items are present in the figure legend, table legend, main text, or Methods section.

n/a	Confirmed	
☐	☒	The exact sample size (*n*) for each experimental group/condition, given as a discrete number and unit of measurement
☒	☐	A statement on whether measurements were taken from distinct samples or whether the same sample was measured repeatedly
☐	☒	The statistical test(s) used AND whether they are one- or two-sided<br>*Only common tests should be described solely by name; describe more complex techniques in the Methods section.*
☒	☐	A description of all covariates tested
☒	☐	A description of any assumptions or corrections, such as tests of normality and adjustment for multiple comparisons
☐	☒	A full description of the statistical parameters including central tendency (e.g. means) or other basic estimates (e.g. regression coefficient) AND variation (e.g. standard deviation) or associated estimates of uncertainty (e.g. confidence intervals)
☐	☒	For null hypothesis testing, the test statistic (e.g. *F*, *t*, *r*) with confidence intervals, effect sizes, degrees of freedom and *P* value noted<br>*Give P values as exact values whenever suitable.*
☒	☐	For Bayesian analysis, information on the choice of priors and Markov chain Monte Carlo settings
☒	☐	For hierarchical and complex designs, identification of the appropriate level for tests and full reporting of outcomes
☒	☐	Estimates of effect sizes (e.g. Cohen's *d*, Pearson's *r*), indicating how they were calculated

Our web collection on statistics for biologists contains articles on many of the points above.

Software and code

Policy information about availability of computer code

Data collection	No software was used for data collection
Data analysis	Kraken v.1.1.1<br>Velvet v.1.2.10<br>guppy barcoder v3.0.3<br>porechop v0.2.3<br>Unicycler v0.4.6<br>PROKKA v1.11<br>ARIBA v.2.14.6<br>SRST2 v.0.2.0<br>SMALT v0.7.4<br>Picard v1.92<br>samtools mpileup v0.1.19<br>bcftools v0.1.19<br>Gubbins v1.4.10<br>snp-sites v.2.5.1<br>RAxML v8.2.8<br>Figtree v1.4.2<br>FastBaps v.1.0.3<br>SNIPPY v.3.0<br>CD-HIT v.4.8.1

TempEST v.1.5.3
BEAST v1.8.4
Tracer v1.7.1
LogCombiner v2.5.0
TreeAnnotator v1.8.4
BRIG v.0.95
blastN v. 2.10.0
Mauve version 2015_02_25
R software

For manuscripts utilizing custom algorithms or software that are central to the research but not yet described in published literature, software must be made available to editors and reviewers. We strongly encourage code deposition in a community repository (e.g. GitHub). See the Nature Portfolio guidelines for submitting code & software for further information.

Data

Policy information about availability of data

All manuscripts must include a data availability statement. This statement should provide the following information, where applicable:

- Accession codes, unique identifiers, or web links for publicly available datasets
- A description of any restrictions on data availability
- For clinical datasets or third party data, please ensure that the statement adheres to our policy

Sequence data that support the findings of this study are available at SRA, accession IDs per isolate are available in Supplementary Data 1 and 3. All data generated during and/or analysed the current study are available from the corresponding author on request. Exchange of biological material should always be in agreement with the local teams.

Human research participants

Policy information about studies involving human research participants and Sex and Gender in Research.

Reporting on sex and gender	NA
Population characteristics	NA
Recruitment	NA
Ethics oversight	NA

Note that full information on the approval of the study protocol must also be provided in the manuscript.

Field-specific reporting

Please select the one below that is the best fit for your research. If you are not sure, read the appropriate sections before making your selection.

☐ Life sciences ☐ Behavioural & social sciences ☒ Ecological, evolutionary & environmental sciences

For a reference copy of the document with all sections, see nature.com/documents/nr-reporting-summary-flat.pdf

Ecological, evolutionary & environmental sciences study design

All studies must disclose on these points even when the disclosure is negative.

Study description	Descriptive genomics study of Salmonella Typhimurium bacterial isolates obtained from bloodstream infections from sub-Saharan Africa. Bacterial isolates were whole genome sequenced and this study includes a maximum likelihood phylogenetic analysis, spatiotermporal phylogenetic analysis, determination of antimicrobial resistance markers and plasmid replicons. For a subset of isolates the phenotypic antimicrobial resistance was determined.
Research sample	Salmonella enterica subspecies enterica serovar Typhimurium isolates originating from bloodstream infections in sub-Saharan Africa. Invasive non-typhoidal Salmonella (iNTS) are the most common cause of bloodstream infections among young children in sub-Saharan Africa, with S. Typhimurium the predominant serovar causing invasive disease. There is a lack of a comprehensive genomic overview of invasive S. Typhimurium from Africa, and our study aimed at filling that gap by gathering all available S. Typhimurium data from sub-Saharan Africa. The sample collection is opportunistic, given that data from iNTS are sparse. The effect of sample bias was considered throughout the manuscript. We have included all available data to reach the most optimal view possible and used rigorous statistical methods to analyse these.
Sampling strategy	The sample set is opportunistic. We aimed to maximally include Salmonella Typhimurium isolates stored from sub-Saharan Africa.
Data collection	A total of 1,420 S. Typhimurium isolates were included in this study, containing 1,303 isolates from Africa. All isolates were Illumina

Data collection

whole genome sequenced and a subset of isolates was sequenced using Oxford Nanopore sequencing.

Of the African isolates, 115 (8.1%) isolates were part of the study by Okoro including the ST313 lineage II D23580 isolate, used here as reference genome.

An additional 816 (57.5%) isolates were sequenced as part of the studies from Van Puyvelde et al., Post et al., Kariuki et al., Feasey et al., Msefula et al., Park et al. and Maclennan et al. Data was recorded following local hospital and laboratory procedures, and as explained in the respective references included in the main manuscript.

As part of this study, 372 (26.2%) additional isolates from sSA were whole genome sequenced. These isolates originated from separate local bacterial surveillance efforts and were recorded following local hospital and laboratory procedures. All available isolates from until 2017 were included.

Of these 372 isolates, 3 isolates originate from ongoing bacterial surveillance in the Centre National Hospitalier Universitaire Hubert Koutougou MAGA of Cotonou, Benin; 33 (2.3%) originate from bloodstream surveillance studies and human reservoir and transmission studies conducted in the Clinical Research Unit of Nanoro (CRUN) in Burkina Faso, 134 (9.4%) originate from the Democratic Republic of Congo (DRC), including ongoing bloodstream surveillance and historical isolates which were bio-banked in the University Hospital Saint-Pierre Brussels (Belgium); 3 (0.2%) isolates originate from returning travellers from Gabon, Guinea and Morocco stored by Sciensano (Belgium); 86 (6%) isolates originate from the Malawi Liverpool Welcome (MLW) bacteraemia archive and dedicated stool samplings by the MLW Research Programme in Blantyre, Malawi; 29 (2.0%) isolates from Nigeria originate from the Community Acquired Bacteremic Syndrome in Young Nigerian Children CABSYNC study and the Community Acquired Pneumonia and Invasive Bacterial Disease (CAPIBD), 69 (4.9%) from Rwanda of which one recent isolate collected by the Rwandan National Reference Laboratory in Kigali (Rwanda) and 68 historical isolates which were bio-banked in the University Hospital Saint-Pierre Brussels (Belgium); 15 (1.1%) isolates originate from population-based surveillance by the Medical Research Council (MRC) Unit The Gambia at the London School of Hygiene & Tropical Medicine (LSHTM) in the Basse region of The Gambia.

We have included the 75 (5.3%) published sequences from Ashton et al., including ST313 sequences isolated by Public Health England in the UK, as context.

Timing and spatial scale

The African Salmonella Typhimurium isolates span 1979 to 2017. The year of isolation is available for all isolates and included in Supplementary Data 1.

The isolates originate from surveillance studies in 19 countries spanning different subregions of Africa (East, Central and West). The country and city information for all isolates is included in Supplementary Data 1.

All available bacterial isolates from the different centers and up to 2017 were included in this study, originating from local bloodstream surveillance efforts.

Data exclusions

No data were excluded from this study

Reproducibility

Phylogenic analyses with RAxML were based on 1000 bootstraps to support the confidence of the tree topology.

Randomization

All available bacterial isolates were included in this study.
For the evolutionary context analysis, a random selection of 10 isolates per invasive S. Typhimurium clade was included.

Blinding

The data did not origin from a clinical trial involving a treatment. Patient data was anonymised. All available bacterial isolates were included in the study.

Did the study involve field work? ☒ Yes ☐ No

Field work, collection and transport

Field conditions

Low-income countries, tropical climate, rainy and dry season alternating and dependent on location above and below the Equator.

Location

See information on data collection above.

Access & import/export

Ethical approval for the Microbiological Surveillance was granted by the Institutional Review Board of the Institute of Tropical Medicine, Anwerp (ref. 613/08, 23/03/2021 and ref. 1108/16, 23/08/2016), by the Ethics Committees of the Antwerp University Hospital, Belgium (ref. 08172613, 01/04/2021 and ref. 16/34/347, 09/01/2017) and the School of Public Health in Kinshasa, DRC (ref. ESP/CE/092/2021, 12/05/2021), University of Malawi College of Medicin Research Ethics Committee (COMREC P.06/20/3071), the Rwanda National Ethics Committee (ref. 903/RNEC/2018, 17/12/2018), the Comité Local d'Ethique Pour la Recherche Biomédicale de Parakou, Benin (0195/CLERB-UP/P/SP/R/SA), the Gambia Government/Medical Research Council Unit The Gambia Joint Ethics Committee (ref. 1087). Research ethical approval was granted by the Ethical Review Boards of the Centre de Recherche en Science Naturelle (CRSN) of Lwiro, DRC and the Kenya Medical Research Institute Scientific and Ethics Review Unit (KEMRI/SERU)-KEMRI/SERU/CGHR/005/3055, the Ethics committee of the Federal Capital Territory FHREC/2012/01/11/16-05-12 and the Ethics committee of Aminu Kano Teaching Hospital- NHREC/21/08/2008/AKTH/EC/1633 and the Health Services Management Board of Kano State- 2/1437AH-9/12/2015.

Disturbance

Occasional (short) stock ruptures (blood culture bottles) as well as canceling of mutual visits due to security issues

Reporting for specific materials, systems and methods

We require information from authors about some types of materials, experimental systems and methods used in many studies. Here, indicate whether each material, system or method listed is relevant to your study. If you are not sure if a list item applies to your research, read the appropriate section before selecting a response.

Materials & experimental systems

n/a	Involved in the study
☒	☐ Antibodies
☒	☐ Eukaryotic cell lines
☒	☐ Palaeontology and archaeology
☒	☐ Animals and other organisms
☒	☐ Clinical data
☒	☐ Dual use research of concern

Methods

n/a	Involved in the study
☒	☐ ChIP-seq
☒	☐ Flow cytometry
☒	☐ MRI-based neuroimaging
